# The Lived Experience of Resilience in Parents of Children With Cancer: A Phenomenological Study

**DOI:** 10.3389/fped.2022.871435

**Published:** 2022-05-30

**Authors:** Yuanhui Luo, Ho Cheung William Li, Wei Xia, Ankie Tan Cheung, Laurie Long Kwan Ho, Joyce Oi Kwan Chung

**Affiliations:** ^1^Xiangya School of Nursing, Central South University, Changsha, China; ^2^Nethersole School of Nursing, The Chinese University of Hong Kong, Hong Kong, China; ^3^School of Nursing, Sun Yat-sen University, Guangzhou, China; ^4^School of Nursing, Hong Kong Polytechnic University, Hong Kong, China

**Keywords:** cancer, children, parents, qualitative study, resilience

## Abstract

**Background:**

Resilience is vital in parents of children with cancer as it can promote parental well-being and minimize maladaptation in the face of the children's cancer. Although existing quantitative studies investigated the influence factors of resilience in the parents, it has not been fully explored about the factors contributing to the resilience of parents and how they respond to and cope with their children's cancer.

**Objective:**

To investigate the lived experience of resilience in the parents of children with cancer from a qualitative perspective to complement existing findings in quantitative studies.

**Methods:**

A phenomenological approach was used. Purposive sampling was performed to recruit parents of children with cancer from two tertiary hospitals in mainland China, followed by one-to-one semi-structured interviews. All of the interviews were audio-recorded and data were analyzed using thematic analysis.

**Results:**

Twenty-three parents, comprising 15 mothers and eight fathers, of children with cancer participated in the interview. Four themes were identified: positive and negative experiences of their children's disease, going through hardships, perceived competence and perceived social support. The most prominent facilitating factor of resilience was the presence of positive attitudes toward the children's cancer, while low level of confidence was the main obstacle.

**Conclusion:**

This study identified certain factors that affect resilience in parents of children with cancer. The findings of this study provide important implications for the development of targeted resilience training programs to enhance resilience in parents of children with cancer. It is crucial for future interventions to focus on cultivating parental resilience to promote parents' mental well-being and improve their quality of life.

**ClinicalTrials.gov ID:**

NCT03631485; URL: https://clinicaltrials.gov/ct2/show/NCT03631485.

## Introduction

An estimated 300,000 new cases of cancer are diagnosed among children aged 0–19 years every year, with the worldwide incidence of childhood cancer increasing constantly (1). Following a child's diagnosis of cancer, the parents have to reorganize the family roles and routines, prepare for the high medical expenses and manage intensive treatment regimes, all of which can cause tremendous stress for the parents (2, 3). Previous studies have shown that parents experience varying degrees of psychological distress during their children's cancer experience and have reported experiencing depressive symptoms, anxiety and post-traumatic stress disorder (4, 5). However, some parents have shown exceptional strength and resilience in response to their children's cancer diagnosis and during the course of treatment (6).

The American Psychological Association defines resilience as the process of adapting well to adversity or trauma (7). Resilience research aims to understand why some people do not develop stress-related disorders, despite being subjected to the same type of adversities that cause such disorders in others (8). Following the postmodern paradigm shift from a problem-oriented approach to one that nurtures strengths, resilience has been increasingly used to identify personal and interpersonal virtues and strengths that can be harnessed to promote individual growth under detrimental conditions (9). Instead of attempting to treat stress-related disorders after individual suffering and economic costs have already been incurred, resilience research attempts to prevent and protect individuals from developing such disorders in the first place (10). Therefore, the development of resilience is an important alternative strategy to promote mental health (11).

Resilience is important in parents of children with cancer as it can minimize maladaptation in the parents by cultivating personal strengths (12). Resilience has been found to benefit the physical and psychological health, especially mental health, of parents of children with cancer and has also been shown to be negatively correlated with depressive symptoms and post-traumatic stress disorder (5, 13). Such stress-related disorders might result in impaired clinical outcomes like suicidality with the influence of neurobiological factors and affective temperaments (14, 15). A recent cross-sectional study exploring the relationship between resilience and the quality of life of parents of children with cancer also found that higher levels of resilience were associated with a better quality of life (16). Compared to parents with low levels of resilience, those with high levels of resilience reported experiencing less psychological distress and better psychosocial functions, such as positive coping and more perceived social support (17, 18). Although existing quantitative studies have found a few influence factors of resilience of parents, such as the above mentioned coping and social support, it has not been fully explored about the factors contributing to the resilience of parents and the responses of and coping mechanisms used by the parents during their children's cancer journey. According to the resilience framework of Kumpfer (19), internal resiliency factors within individuals—cognitive, emotional, spiritual, behavioral, and physical factors—represent fundamental elements that are essential for adapting well in the face of adversities. Individuals considered more resilient have significantly more of these internal resiliency factors. However, a review of existing literature revealed a paucity of studies on what resiliency factors the parents have or lack and how they apply these internal competencies to face with their children's cancer. A thorough understanding of such factors is a prerequisite for the development of appropriate interventions to boost parental resilience, facilitate the development of better coping mechanisms to deal with the stress caused by their children's cancer, and enhance their quality of life. This qualitative study aimed to explore the factors that facilitate and hinder resilience in parents of children with cancer.

## Materials and Methods

### Study Design and Participants

A qualitative study was conducted in two tertiary hospitals in mainland China between August 2018 and November 2018 using a qualitative approach to explore the factors contributing to resilience in parents of children with cancer. This study is reported in accordance with the Standards for Reporting Qualitative Research (20).

The sample for the present study was selected from the participants of a previous cross-sectional study (16) that examined the level of resilience in parents of children with cancer. The inclusion criterion was primary caregivers, either fathers or mothers, of children (0–19 years) with cancer. The parents with cognitive impairment identified in medical records were excluded. To gain a thorough understanding of the factors that contribute to low parental resilience compared to the factors that contribute to high parental resilience, we divided the sample (16) into two groups: low resilience (scores of 0–49) and high resilience (scores of 50–100), as measured using the Connor-Davidson Resilience Scale (CD-RISC) (21). These categories were based on population data (22) on CD-RISC showing a mean score of 50 in Chinese subjects exposed to severe stress. The CD-RISC consists of 25 items and utilizes a 5-point Likert scale. The total score ranges from 0 to 100, with higher scores reflecting higher levels of resilience. Participants from these two groups were then randomly selected and invited to participate in a one-to-one semi-structured interview; during selection, one participant was randomly selected from the high resilience group, following which another participant was randomly selected from the low resilience group. Recruitment for the interviews continued until the research committee agreed that the collected data no longer yielded new information or allowed for further coding.

### Data Collection

To conceptualize and clarify specific areas of the phenomenon this qualitative study explored, the resilience framework was used to inform the inquiry process. Based on the internal resiliency factors in the resilience framework, an interview guide was developed by the research committee, which comprised the head of a pediatric oncology department, a professor of psycho-oncology, two senior pediatric oncology nurses and two postgraduate students trained in qualitative studies. Three main areas were mentioned in the interview guide: (1) overall feelings about and perceptions of the child's cancer, (2) perceived abilities and behaviors in solving problems, (3) perceived support from others. Open-ended research questions and probing questions were used. The key questions included in the interview guide are listed in the Appendix as Supplementary Material.

Eligible parents of children with cancer who expressed their willingness to participate in the interview through a previous questionnaire were contacted by phone and invited to participate in a one-to-one semi-structured interview. All interviews were conducted face-to-face in private counseling rooms in the ward and were audio-recorded with the participants' consent. Each interview lasted 30–40 min. Before the interview, the researcher gave a brief self-introduction and explained the objectives of the interview to the participants. Confidentiality was guaranteed, and the participants had the right to end the interview at any time if and when they felt uncomfortable.

### Data Analysis

NVivo 11 software (2015; QSR International, Melbourne, Australia) was used to organize and manage the qualitative data (23). A phenomenological approach was followed to understand and illuminate the factors that affect resilience in parents of children with cancer. Researchers firstly bracketed out personal prejudices and any presuppositions, such as personal and theoretical knowledge about factors that influence resilience. Then intuition followed, where researchers avoided criticism, evaluation or opinion on matters submitted by participants in the interview process. The following stages were analysis and description. Thematic analysis outlined in Braun and Clark's method of qualitative data analysis were used in this study (24). All interview recordings were transcribed verbatim and double-checked for accuracy. First, the transcripts were read multiple times to familiarize the researchers with the data, enabling them to record their initial ideas. Second, the researchers identified statements that were directly relevant to the factors contributing to parental resilience and performed coding. Third, all codes were gathered and categorized into potential sub-themes, which were then combined into themes. Fourth, the themes were reviewed by checking their coherence with coded data extracts and their relationship to the entire dataset. Fifth, the themes were defined and named. A clear name and description for each theme were generated in this phase and the ongoing refining of the themes was performed for further analyses. Finally, report was produced. Vivid and convincing examples of data extracts were selected and analyzed in the light of the research questions to generate the report.

A phenomenological attitude is crucial in a phenomenological approach. In order to ensure the integrity of the phenomenological attitude, investigators reflected on personal and theoretical knowledge of factors contributing to resilience and reported any predispositions that might have affected the data interpretation and presentation of the findings prior to the start of the study. Team meeting was also held regularly to monitor the study quality, in which the investigators presented reflections on decision-making, theme emergence and personal responses throughout interviewing and data analysis, and the research committee reviewed the interview transcripts to ensure that the phenomenological attitude was maintained.

Several strategies were used to ensure the trustworthiness of the study. First, to enhance credibility, two researchers independently analyzed the qualitative data. To reach a consensus on the data, all of the inconsistent codes and themes were discussed by the research committee. Member checking was also conducted to verify the accuracy of the recordings, wherein the transcripts of the interviews were returned to the participants. Second, the triangulation method was used to strengthen confirmability and reduce the effect of investigator bias. An experienced researcher interviewed the investigators to identify any preconceptions that might prejudice their interpretations of the data. Third, to improve transferability, detailed descriptions and contextual information were provided. Finally, to ensure dependability, a detailed research process was reported to enable future researchers to reproduce the work.

### Ethical Considerations

This study was approved by the Institutional Review Board of the University of Hong Kong/Hospital Authority Hong Kong West Cluster (No. UW 18-371). Written informed consent was obtained from the participants at the beginning of the study. This informed consent provided detailed information about the purpose and procedure of the study, the right to withdraw at any time without reprisal and the confidentiality of personal information.

## Results

Twenty-three participants completed the interview. Of these, 11 participants were from the low resilience group and 12 were from the high resilience group. The participants were mostly female (65.2%) and married (91.3%), with a mean age of 33.3 years (standard deviation [SD] = 5.1 years). Most of the children (69.6%) were diagnosed with hematological cancers, and the remaining 30.4% were diagnosed with solid tumors. The children had a mean age of 6.4 years (SD = 4.0 years), and the majority of them had been diagnosed with cancer <1 year before the time of conducting this study (95.7%). The characteristics of the participants are presented in Table 1.

**Table 1:** Characteristics of parents and children (*N* = 23).

	***N* (%)**
**Parents characteristics**	
Age of parents, mean (SD), years	33.3 (5.1)
**Sex of parents**	
Female	15 (65.2)
Male	8 (34.8)
**Marital status**	
Married	21 (91.3)
Divorced	2 (8.7)
**Educational attainment**	
Primary school	3 (13.0)
High school	17 (73.9)
College	3 (13.0)
**Income (**¥**, CNY** ^**a**^**)**	
<3,000	14 (60.9)
3,000–5,000	8 (34.8)
>5,000	1 (4.3)
**Children characteristics**	
Age of children, mean (SD), years	6.4 (4.0)
**Sex of children**	
Female	8 (34.8)
Male	15 (65.2)
**Type of cancer**	
Hematology tumor	16 (69.6)
Solid tumor	7 (30.4)
**Type of treatment**	
Single therapy	20 (87.0)
Multiple therapies	3 (13.0)
**Time since diagnosis (month)**	
0–6	18 (78.3)
7–12	4 (17.4)
>12	1 (4.3)

^a^* CNY, China Yuan; US$ 1.00, ¥ 7.02*.

Four themes were identified as reflecting the parental lived experience in resilience: positive and negative experiences of their children's disease, going through hardships, perceived competence and perceived social support. The themes, subthemes and examples of quotes from the interviews are shown in Table 2.

**Table 2:** Themes, subthemes and examples of quotes from interviews.

**Themes**	**Subthemes**	**Examples of quotes**
Theme 1: Positive and negative experiences of their children's disease	Subtheme 1: Being positive and optimistic	“After knowing about the disease, I slowly think of it as a cold that happens every month. No big deal. I don't think too seriously; it is just a little bit worse than a cold.” *Parent 1, female, child aged 5 years*. “My husband hopes that I will take care of the child at home. Then he works to make money to maintain our daily life. After ending the treatment, my son will go to school like normal children; life will be better.” *Parent 3, female, child aged 3 years*. “It's a change in life attitudes. I used to want to buy a good car and a good house, the child should go to a good school… Now I think the child's health is the most important. Being healthy is enough.” *Parent 4, female, child aged 1 year*.
	Subtheme 2: Feeling powerless	“In fact, I don't know how to take care of my child because I'm not patient… Now the price of goods has risen too high. I really don't have the ability; I'm not smart enough and really incompetent in making money.” *Parent 17, male, child aged 12 years*. “I feel that I'm experiencing something abnormal. My peers don't experience these things. They work smoothly and live a better life than me. Compared to them, I'm a little inferior.” *Parent 14, male, child aged 8 years*. “Nothing was difficult for me before, but I don't know what to do now. This time I don't know what to do, I can't think of any solutions… She (the child) suffers from this disease, I can't do anything.” *Parent 16, female, child aged 6 years*.
Theme 2: Going through hardships	Subtheme 1: Problem-focused approach	“They (other parents) have more experience because they have been here (the hospital) longer. Sometimes I learn from them about how to care for the child, and consult them, especially about the child's diet.” *Parent 9, female, child aged 4 years*. “About this disease, we must accept the reality and find ways to deal with it. You should actually do something (to solve problems), not just worry. You are the head of the family. Just like the house pillar, if you fall down, the whole house will collapse.” *Parent 10, male, child aged 11 years*.
	Subtheme 2: Emotion-focused approach	“He (the child) has a stubborn temper; sometimes, I can't make any sense of him. I become crazy when he makes trouble… I usually leave him in the ward, come out to breathe and then go back.” *Parent 2, female, child aged 5 years*. “Since diagnosis, I've had no contacts with them (previous friends); I felt that I did not know what to say when they talked to me. I don't like to go out and talk to others. I just play with the phone and sleep when tired; I do not communicate with others.” *Parent 13, female, child aged 8 years*.
Theme 3: Perceived competence	Subtheme 1: Being competent	“When he (the child) was vomiting, he did not want to eat anything. Then I cooked porridge and beans, made them into a paste with a soymilk machine… I cooked various foods, he ate a little of each food, which was what I wanted. If I just cook one type of food, he might not eat anything.” *Parent 11, female, child aged 13 years*. “To treat this disease, the most important thing is that the child is willing to cooperate. If she (the child) does not cooperate, it is useless if people around are anxious. Then the first step is to care about her feelings and calm her down… I always comfort her by saying ‘Daddy's here, I can solve everything for you'.” *Parent 7, male, child aged 14 years*. “When the platelets were good, I took him out for a walk. Because his legs were not strong yet, we did not walk too fast. I wouldn't let him stay in the room for the whole day. Sometimes I took him to play table tennis.” *Parent 11, female, child aged 13 years*.
	Subtheme 2: Ineffective parent-child communication	“When he (the child) was asked to wear a mask, he just wore it for a while. It did not take a long time for the mask to start falling again, at which point I yelled at him. I used a very harsh way to deal with it. I feel like I know no good way to communicate with him.” *Parent 13, female, child aged 8 years*.
Theme 4: Perceived social support	Subtheme 1: Being grateful	“Whenever I feel stressed, I want to speak out, I talk to his (the child's) father. I feel better after sharing. We support each other, and I feel that the closest people are family.” *Parent 6, female, child aged 2 years*. “The support of family and friends is substantial… financial, material resources, or spiritual… I feel very fortunate to have such good relatives and friends who help and support my family; I deeply appreciate it.” *Parent 9, female, child aged 4 years*.
	Subtheme 2: Perceived inadequate social support	“The neighbors did not allow their children play with my child. They may feel that my child is an inauspicious person. I think there's no need to explain anything to them. I get used to it gradually, too many people treat you with this kind of ‘colored eyes'.” *Parent 14, male, child aged 8 years*

### Theme 1: Positive and Negative Experiences of Their Children's Disease

#### Subtheme 1: Being Positive and Optimistic

More than half of the parents in the high resilience group showed a positive cognition of and optimistic attitudes toward their children's cancer. They regarded the children's disease as being akin to a relatively serious cold that happens once a month and did not worry much about it. Some parents reported that they believed in advanced medical technology and were hopeful about the child's prognosis. Most of them had positive plans for the future, having arranged schooling for the child in advance and finding new jobs to resolve financial issues. Moreover, most parents made their children's cancer treatment a priority and regarded health to be the most important thing in life. They reflected that their children's disease had changed their attitudes toward life and that they were no longer preoccupied by trivial worries. The parents cherished their current lives even more than before their children's cancer diagnosis and were satisfied with their usual daily lives.

Parent 1 (Female, child aged 5 years): After knowing about the disease, I slowly think of it as a cold that happens every month. No big deal. I don't think too seriously; it is just a little bit worse than a cold.

#### Subtheme 2: Feeling Powerless

More than half of the parents in the low resilience group reported inadequate confidence in the face of their children's cancer. Some of them doubted their own abilities to take care of their children and expressed a sense of powerlessness in the face of financial issues. A few parents felt inferior to their peers. They reflected that their lives were seriously disrupted and that they were frustrated with their inability to do anything in the face of their children's cancer.

Parent 17 (Male, child aged 12 years): In fact, I don't know how to take care of my child because I'm not patient… Now the price of goods has risen too high. I really don't have the ability; I'm not smart enough and really incompetent in making money.

### Theme 2: Going Through Hardships

#### Subtheme 1: Problem-Focused Approach

Some parents in the high resilience group confronted their children's disease courageously and dealt positively with the problems encountered when caring for their children. They actively learned about their children's disease from health care professionals and carefully read the care manuals distributed by the hospital. When they encountered problems when caring for their children, they consulted other veteran parents who had had children with cancer and sought support from their families. Some fathers took the initiative to support their families. They worked hard to make more money and tried their best to raise money to meet the high expenses of the children's treatment. A few of them even gave up smoking and drinking to save money. The fathers fulfilled a core role in their respective families and considered themselves to be responsible for supporting the entire family.

Parent 9 (Female, child aged 4 years): They (other parents) have more experience because they have been here (the hospital) longer. Sometimes I learn from them about how to care for the child, and consult them, especially about the child's diet.

#### Subtheme 2: Emotion-Focused Approach

Many parents reported experiencing negative emotions when their children were diagnosed with cancer. Depressive symptoms and anxiety were the most common emotions experienced by the parents. They were also easily irritated due to the demands of caring for their children or any misbehaviours on the part of the children. These parents usually managed their emotions by exercising self-control practices, such as leaving a stressful scene to calm down. In addition, some parents in the low resilience group showed avoidance behaviors after their children were diagnosed with cancer. They were unwilling to communicate with others and had less contact with their friends. During the children's treatment in the hospital, they seldom communicated with other parents of children with cancer in the same ward and preferred to stay alone.

Parent 13 (Female, child aged 8 years): Since diagnosis, I've had no contacts with them (previous friends); I felt that I did not know what to say when they talked to me. I don't like to go out and talk to others. I just play with the phone and sleep when tired; I do not communicate with others.

### Theme 3: Perceived Competence

#### Subtheme 1: Being Competent

Many parents in the high resilience group showed high competence in caring for their children with cancer. They not only cared about the physical health, but also paid attention to the mental status of the children. Some parents reported that they became more patient after their children were diagnosed with cancer. They were attentive to special dietary requirements and tried various methods by which to improve their children's appetites. Some parents realized that it was important to focus on the children's emotions and attitudes toward treatment, especially in the case of teenagers, prompting the parents to encourage and comfort their children. Moreover, some parents thought that physical activity was necessary for and aided their children's recovery. They companied their children during outdoor activities, sanctioned based on the children's conditions. If the children could not go out due to treatment constraints, the parents accompanied the children in practicing handicrafts or painting in the ward.

Parent 7 (Male, child aged 14 years): To treat this disease, the most important thing is that the child is willing to cooperate. If she (the child) does not cooperate, it is useless if people around are anxious. Then the first step is to care about her feelings and calm her down… I always comfort her by saying “Daddy's here, I can solve everything for you”.

#### Subtheme 2: Ineffective Parent-Child Communication

Some parents in the low resilience group reflected that they did not know how to communicate with their children, especially when dealing with misbehaviours or unstable emotions caused by the disease. The parents sometimes punished misbehaviours using verbal reprimands. Although these parents realized that verbal reprimands were undesirable, they had no effective ways of communicating with the children to correct their misbehaviours.

Parent 13 (Female, child aged 8 years): When he (the child) was asked to wear a mask, he just wore it for a while. It did not take a long time for the mask to start falling again, at which point I yelled at him. I used a very harsh way to deal with it. I feel like I know no good way to communicate with him.

### Theme 4: Perceived Social Support

#### Subtheme 1: Being Grateful

Many parents reported that they received a lot of social support when coping with their children's cancer and considered partner support to be the most important component of external support. Mutual partner support was most strongly reflected by parents in the high resilience group. These parents shared the responsibility of taking care of their children, relieved stress by talking to each other and gave each other comfort when required. Some parents also expressed their appreciation for financial and spiritual support from friends, relatives and society. These parents reported that it would be impossible for them to tide over their difficulties without the support of others. They were satisfied with and grateful for the support.

Parent 9 (Female, child aged 4 years): The support of family and friends is substantial… financial, material resources, or spiritual… I feel very fortunate to have such good relatives and friends who help and support my family; I deeply appreciate it.

#### Subtheme 2: Perceived Inadequate Social Support

Some parents in the low resilience group reflected that little support was available in the face of their children's cancer. The families merely blamed the parents for the disease while not providing any support in taking care of the children. A few parents reported that the cancer resulted in their children being misunderstood and isolated by others. They thought that strangers looked at their children through ‘colored eyes' and that their children were isolated by other children due to a negative perception of cancer.

Parent 14 (Male, child aged 8 years): The neighbors did not allow their children play with my child. They may feel that my child is an inauspicious person. I think there's no need to explain anything to them. I get used to it gradually, too many people treat you with this kind of “colored eyes”.

## Discussion

This study identified various factors that facilitate or hinder resilience in parents of children with cancer. The most prominent factor that facilitated parental resilience was the presence of positive and optimistic attitudes toward the children's cancer. One previous study has shown that optimism is positively associated with resilience in other populations such as university students (25). Another study reported that optimism is a trait of resilient parents; it plays a major role in their adaptation to stressful conditions and is the most influential factor that tempers the effects of life stressors (26). Interventions that aim to cultivate positive cognition and optimism can thus be developed to enhance resilience in parents of children with cancer. This study also showed that a problem-focused approach in dealing with their children's cancer facilitated resilience in parents. This was consistent with the findings of other studies that emphasized the positive role of problem-focused coping in promoting the psychological well-being of parents of children with cancer (27, 28). Problem-focused coping usually involves directly confronting stressors and generating possible solutions to a problem. It has been observed that training programs that develop problem-solving skills can alleviate depressive symptoms and anxiety in the mothers of children with cancer, especially for those whose children have recently diagnosed (29). Interventions that incorporate training to develop problem-solving skills may thus promote resilience in the parents of children with cancer. These findings were also in coincidence with the commonalities across resilience models for parents of children with chronic health conditions, which emphasized the influence of cognitive appraisals and coping strategies in parental resilience (30).

Parents with high levels of resilience also showed high competence in taking care of the sick children. Extant literature has documented high-quality caregiving behaviors in the parents and shows that such behaviors alleviate distress in the children (31). It is therefore necessary to help parents build the caregiving skills required to navigate the needs of children with cancer. The findings of this study also showed that perceived financial and spiritual support contribute to parental resilience, consistent with a previous study that reported that perceived social support is positively associated with resilience in the parents of children with cancer (32). In this study, parents in the high resilience group were more inclined to perceive support from others and show their appreciation. Future interventions can therefore focus on cultivating gratitude in parents to enhance their resilience.

More than half of the parents in the low resilience group reported inadequate confidence in the face of their children's cancer, which was a major obstacle to promoting parental resilience. Extant quantitative studies have revealed low levels of self-efficacy in the parents of children with cancer (33, 34). Character strength training, which can help individuals determine and harness the advantageous traits they possess, has shown to positively influence resilience in other populations (35, 36). Therefore, such training can be integrated into future interventions to bolster confidence in parents when caring for children with cancer and thereby enhance resilience. A lack of effective parent-child communication skills was another factor that hindered resilience in the parents. Given the integral role of parent-child communication in promoting a positive outlook in children and the leading roles that parents play in such relationships (37), targeted parent-child communication skills training is necessary to improve the adjustment and well-being of both parents and their children with cancer. In addition, this study revealed the presence of negative emotions such as depressive symptoms and anxiety in many parents, some of whom resorted to emotion-focused coping mechanisms to regulate their negative emotional reactions to their children's cancer. Although emotion-focused coping mechanisms such as self-control is the preferred method of stress management among the Chinese population (38, 39), other avoidance behaviors such as retreating from interpersonal relationships were also exhibited by some parents in this study. Considering the benefits of interpersonal interaction to parental well-being (40), problem-focused coping mechanisms such as consulting other parents of children with cancer can help parents to better cope with the situation. This would improve parental resilience and psychological well-being.

A phenomenological approach can produce rich data to provide a profound, detailed understanding of the factors contributing to resilience in parents of children with cancer. However, one limitation in the process of data collection was the use of a framework, which might direct what arose during the interviews. Another limitation of this study is that it focused on individual internal factors that contribute to resilience in the parents of children with cancer and did not comprehensively explore external environmental factors such as community. Given that the environmental context can affect parental resilience, future studies should explore protective and risk factors arising from the environmental context as a complement to better understand the factors influencing resilience in the parents of children with cancer.

### Implications for Future Research and Practice

The findings of the present study are important for the development of targeted resilience training programmes to enhance resilience in the parents of children with cancer in the near future. It is crucial for future interventions to focus on cultivating parental resilience to promote their mental well-being and improve their quality of life. In addition, given the different points of contact with parents of children with cancer, it is critical for all health care practitioners to understand the factors contributing to parental resilience and then develop targeted resilience training programmes in collaboration with other professionals. For example, given that parent-child communication skills were found to facilitate parental resilience, in the process of health education, health care practitioners should make parents aware of the importance of communication. Corresponding knowledge classes may be provided to teach parents how to communicate with their children with cancer, such as how to listen to their children and share their own feelings. Equipping parents with caregiving skills is another effective way to improve resilience in the parents of children with cancer. In addition, to emphasize comprehensive physical and mental healthcare for children during hospital visits, health care practitioners should implement a long-term follow-up program to determine whether parents lack certain care skills, and then provide the appropriate health education. Finally, health care practitioners should pay more attention to parents with low levels of resilience and implement targeted resilience training to improve their well-being.

## Conclusion

This study addressed an existing gap in the literature by evaluating the factors that contribute to resilience in the parents of children with cancer. Given that we identified various factors that facilitate and hinder resilience in parents of children with cancer, our findings provide important implications for the development of targeted resilience training programmes to enhance parental resilience and promote their well-being. Specifically, health care professionals could encourage the parents to focus on positive things around them to foster positive emotions and optimism. It is also highly recommended to integrate parent-child communication skills, problem-solving skills and caregiving skills training into the resilience training programme. Parent-child communication skills training would be helpful for the parents while providing psychosocial support for their child with cancer. Problem-solving skills training would enhance the parental resilience by cultivating positive coping strategies. Helping parents build the caregiving skills required to navigate the needs of children with cancer would benefit both parents and their children.

## Data Availability Statement

The raw data supporting the conclusions of this article will be made available by the authors, without undue reservation.

## Ethics Statement

The studies involving human participants were reviewed and approved by the Institutional Review Board of the University of Hong Kong/Hospital Authority Hong Kong West Cluster (No. UW 18-371). The patients/participants provided their written informed consent to participate in this study.

## Author Contributions

Material preparation, data collection, and analysis were performed by YL, HL, and WX. The first draft of the manuscript was written by YL. All authors commented on previous versions of the manuscript. All authors read and approved the final manuscript and contributed to the study conception and design.

## Conflict of Interest

The authors declare that the research was conducted in the absence of any commercial or financial relationships that could be construed as a potential conflict of interest.

## Publisher's Note

All claims expressed in this article are solely those of the authors and do not necessarily represent those of their affiliated organizations, or those of the publisher, the editors and the reviewers. Any product that may be evaluated in this article, or claim that may be made by its manufacturer, is not guaranteed or endorsed by the publisher.
