# Supplementary material for: The Lived Experience of Resilience in Parents of Children With Cancer: A Phenomenological Study
Source: Front Pediatr. 2022 May 30;10:871435. doi: 10.3389/fped.2022.871435 (PMC9189362; doi:10.3389/fped.2022.871435)
Supplement: Supplementary file 1 [file Table_1.DOCX]

**Appendix 1** The interview guide

1. Could you tell me about your feelings after learning that your child had cancer?
2. How did you manage your emotions after learning that your child had cancer?
3. What are your attitudes towards your child’s cancer?
4. How do you appraise your ability to take care of your child with cancer?
5. What do you usually do when you encounter difficulties in life?
6. What kinds of plans or expectations do you have for the future?
7. What support have you received in dealing with your child’s cancer?
